# Low Levels of Empathic Concern Predict Utilitarian Moral Judgment

**DOI:** 10.1371/journal.pone.0060418

**Published:** 2013-04-04

**Authors:** Ezequiel Gleichgerrcht, Liane Young

**Affiliations:** 1 Institute of Cognitive Neurology (INECO), Buenos Aires, Argentina; 2 Psychology Department, Boston College, Chestnut Hill, Massachusetts, United States of America; 3 Institute of Neurosciences, Favaloro University, Buenos Aires, Argentina; 4 Laboratory of Neurosciences, Diego Portales University, Santiago, Chile; The University of Queensland, Australia

## Abstract

Is it permissible to harm one to save many? Classic moral dilemmas are often defined by the conflict between a putatively rational response to maximize aggregate welfare (i.e., the utilitarian judgment) and an emotional aversion to harm (i.e., the non-utilitarian judgment). Here, we address two questions. First, what specific aspect of emotional responding is relevant for these judgments? Second, is this aspect of emotional responding selectively reduced in utilitarians or enhanced in non-utilitarians? The results reveal a key relationship between moral judgment and *empathic concern* in particular (i.e., feelings of warmth and compassion in response to someone in distress). Utilitarian participants showed significantly reduced empathic concern on an independent empathy measure. These findings therefore reveal diminished empathic concern in utilitarian moral judges.

## Introduction

Recent work in psychology and neuroscience has aimed to identify the cognitive and neural processes that support moral cognition [1], [2], including emotional responding, abstract reasoning, and the processing of norms [3]–[6]. More specifically, a number of studies support the role of emotions in moral judgment [4], [7]–[9] and in particular a dual-process model of moral judgment [10]–[15]. On this model, both automatic emotional processes and controlled cognitive processes drive moral judgment. For example, when people must choose whether to harm one person to save many, emotional processes typically support one type of response (e.g., don’t harm the individual), while controlled processes support a different response type (e.g., save the greatest number of lives).

According to prior research [13], [15], these processes are also engaged differently depending on the nature of the scenario in question. When people encounter an *impersonal* dilemma, which lacks salient emotional content (e.g., would you *turn a trolley* away from five people and onto one person?), most people endorse harming the one person for the greater good, thereby delivering the *utilitarian* response. By contrast, when people are presented with a *personal* dilemma (e.g., would you *push a man* in front of a trolley so that his body stops the trolley from hitting five people?), emotions are engaged, leading the majority of responders to reject the harmful act, thereby delivering a *non-utilitarian* response. Recent amendments to dual-process models of moral judgment suggest further that personal (as opposed to impersonal) harms are more precisely defined by the interaction between *intended* harm and harm via *personal force*, i.e. the execution of a motor act that involves using one’s own physical means to harm someone [16], [17]. Such personal harms (intended harms via personal force), a focus of the current paper, typically elicit the most robust emotional responses and therefore non-utilitarian judgments.

Convergent evidence using behavioral and neuropsychological approaches suggests that emotional deficits (e.g., alexythimia, the inability to articulate one’s emotional experience) or otherwise disrupting emotional processes leads to more utilitarian moral judgment [15], [18]–[21]. In one recent study, participants with higher scores on measures of antisocial personality (and, presumably, disrupted emotional processes) were more likely to endorse utilitarian options in moral dilemmas [3]. Conversely, enhanced emotional processing among neurotypical participants has led to the greater condemnation of harmful (and, occasionally, harmless) acts in a series of studies. For example, priming participants to experience disgust via hypnosis [22], exposing participants to a bitter taste [23] or a disgusting smell [5], or even seating participants at a dirty desk [24] resulted in harsher moral judgments. In fact, even self-reported measures of one’s proneness to feel disgusted have been associated with harsher judgments, highlighting the impact of emotion on moral cognition [25]–[27]. Meanwhile, “disrupting” controlled processing by imposing a cognitive load on participants was found to slow down utilitarian judgments [11], while pressuring participants to deliver judgments to moral scenarios more quickly (without deliberate reflection) led to a greater proportion of deontological responses [28].

Is utilitarian judgment, among neurotypical participants, in the absence of behavioral primes, simply the result of “enhanced cognition” (e.g., better cognitive control, abstract reasoning), or also reduced emotion? If utilitarian judgment is associated with reduced emotion as suggested by the neuropsychological evidence, what specific aspect of emotional responding is at stake? The present study seeks to address these questions in neurotypical participants by identifying the key components of emotional processing for moral judgments. In addition, the present study examines whether utilitarian responders are capable of endorsing killing one to save many because they are *less* emotional than the “average” moral judge, or whether responders who deliver consistently *non*-utilitarian judgments are unwilling or unable to kill one to save many because they are *more* emotional.

We address these questions by focusing on the role of *empathy* in moral judgment. The term empathy has been applied broadly to knowing what others are thinking or feeling (i.e., perspective taking), experiencing concern for another individual (i.e., empathic concern), and even self-oriented feelings that arise when witnessing or caring for others in pain or distress (i.e., personal distress) [29]. To characterize the relationship between moral judgment and empathy, we presented participants with three pairs of personal and impersonal scenarios, in conjunction with independent measures of distinct components of empathy. To foreshadow the results, we found across three experiments that responders who were consistently *utilitarian* showed significantly *lower* levels of empathic concern, in the absence of any other cultural or demographic differences.

## Experiment 1

The experiments in this study were approved by the ethics committee at the Institute of Cognitive Neurology (INECO) according to the principles expressed in the Declaration of Helsinki.

### Participants

Volunteer participants [*n = *1339; mean age: 25.7 years (*SD* = 11.2), mean education: 13.4 years (*SD* = 3.9)] were recruited by word of mouth and directed to the present web-based study. We excluded subjects who (a) were younger than 18 years old, (b) reported a personal history of traumatic brain injury, psychiatric disease, or drug abuse, or (c) failed the “control” question at the end of the experiment, by answering “No” in response to “Did you answer all questions honestly/thoughtfully?” All participants gave informed written consent before beginning the experiment.

### Procedure

Participants reported age, gender, and education on the first page of the online study, and then completed a series of tasks, the order of which was randomized across participants. Descriptions of tasks follow.

#### Moral judgment

Participants were presented with a pair of moral dilemmas, in counterbalanced order (i.e., some participants read the impersonal scenario first, while others read the personal scenario first). Each scenario required participants to choose whether to harm one person to save five people. The “personal” dilemma featured an emotionally salient harm (e.g., pushing a man off a bridge); the “impersonal” dilemma featured a less emotionally salient harm (e.g., flipping a switch to redirect a trolley onto a man) [12], [13]. In particular, participants were presented with the standard trolley dilemma (impersonal) and the footbridge dilemma (personal) (Table S1). In the trolley dilemma, the utilitarian response was to flip the switch to turn the trolley away from five people and onto one person instead, whereas the non-utilitarian response was to allow the trolley to hit the five people. In the footbridge dilemma, the utilitarian response was to push a man off a bridge so that his body would stop the trolley from hitting five people further down the tracks, whereas the non-utilitarian response was to allow the trolley to allow the trolley to hit the five people.

**Figure 1:**
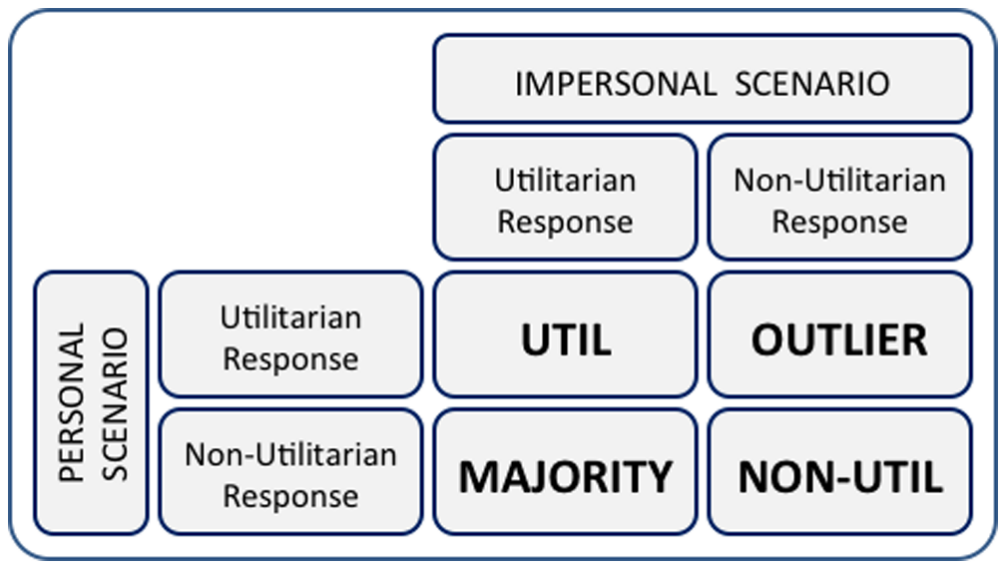
Grouping criteria based on responses to impersonal and personal moral scenarios.

Participants’ responses to the pair of moral dilemmas were used to classify participants into four groups, for analyses below (Figure 1): (1) **UTILITARIAN (UTIL)** participants delivered the utilitarian response for *both* scenarios; (2) **NON-UTILITARIAN (NON-UTIL)** participants delivered the non-utilitarian response for *both* scenarios; (3) **MAJORITY** participants delivered the utilitarian response for the impersonal scenario but the non-utilitarian response for the personal scenario, a response pattern observed in the vast majority of participants across a number of prior studies using the same scenarios and therefore reflecting the “average” or modal moral judge [10], [11], [13], [15], [20], [30], [31]; and (4) **OUTLIER** participants delivered the non-utilitarian response for the impersonal scenario but the utilitarian response for the personal scenario. Of the total number of participants in this analysis, 213 (15.9%) were classified into the **UTIL** group, 505 (37.7%) into the **NON-UTIL** group, 606 (45.3%) were classified into the **MAJORITY** group, and the remaining 15 (1.1%) the **OUTLIER** group.

#### Moral knowledge

Participants completed the Moral Behavior Inventory (MBI) designed by Mendez et al. [20], a 24-item scale presenting situations (e.g., “Fail to keep minor promises”, “Temporarily park in a handicap spot”, and “Falsely get out of jury duty”) to be labeled as “not wrong”, “mildly wrong”, “moderately wrong”, or “severely wrong’’. The MBI aims to measure participants’ ability to distinguish right from wrong, providing a measure of “moral gnosia” [20].

#### Religiosity/Spirituality

Participants completed the Daily Spiritual Experience Scale (DSES) [32] Participants rated fourteen items on the frequency, from 0 (many times a day) to 6 (never or almost never), with which they experience each statement (e.g., “I find strength in my religion or spirituality”, “I ask for God’s help in the midst of daily activities”). In addition, the DSES presents two items rated from 1 (“Not at all close”) to 4 (“As close as possible”) in relation to their desire to be closer to God and how close they feel to God.

#### Empathy

Participants completed the Interpersonal Reactivity Inventory (IRI) [33], a 28-item self-report questionnaire with four 7-item subscales, assessing specific aspects of empathy: empathic concern (the tendency to experience feelings of warmth, compassion, and concern for other people), personal distress (one’s own feelings of personal unease and discomfort in reaction to the emotions of others), perspective taking (the tendency to adopt the point of view of other people), and fantasy (the tendency to transpose oneself into the feelings and actions of fictitious characters). Empathic concern and personal distress represent two independent measures of emotional empathy, while perspective taking and fantasy represent measures of cognitive empathy.

### Results

Given our large sample sizes, between-group differences are reported with *p* values and their associated effect sizes in terms of Cohen‘s *d* scores (cf. Iyer et al. [34]). Following Cohen’s [35] classification of effect sizes, we consider main effects to be statistically significant and relevant with *d* scores >.40 (i.e. moderate effect size or higher).

#### UTIL vs. NON-UTIL comparison

There was no difference between participant groups (UTIL, NON-UTIL, MAJORITY, and OUTLIER) in terms of age (*F*_3,1335_ = 1.57, *p* = .19), gender (*χ* ^2^ = 2.35, *p* = .50, df = 3), education (*F*_3,1335_ = 1.07, *p* = .58), moral knowledge, as measured by the MBI (*F*_3,1335_ = 1.72, *p* = .16), or religiosity, as measured by the DSES (*F*_3,1335_ = 1.56, *p* = .20) (Table 1).

**Table 1:** *Mean* (*SD*) values for demographic variables and data obtained from Experiment 1 with questionnaires measuring moral knowledge, religiosity, and empathy.

		UTIL*n* = 213	NON-UTIL*n* = 505	MAJORITY*n* = 606	OUTLIER*n = 15*	
Age (years)		26.8 (13.3)	25.8 (10.3)	25.3 (11.2)	24.7 (10.3)	
Gender (M : F)		100∶113	228∶277	253∶353	6∶ 9	
Education(years)		13.3 (4.6)	13.6 (3.7)	13.4 (4.2)	13.5 (3.4)	
MBI		60.3 (11.8)	60.0 (12.2)	61.6 (10.3)	56.1 (17.8)	
DSES		58.3 (22.1)	58.4 (22.6)	60.9 (19.9)	57.0 (20.1)	
IRI	Perspective Taking	20.5 (5.6)	20.6 (5.2)	21.0 (5.2)	20.6 (5.2)	
	Fantasy	18.5 (5.9)	18.9 (5.7)	19.5(5.6)	18.9 (5.8)	
	Empathic Concern	20.5 (6.4)	24.2 (5.5)	24.7 (5.5)	24.2 (5.5)	**
	Personal Distress	14.6 (4.8)	14.8 (4.7)	14.9 (4.5)	14.8 (4.7)	

** *F*_3,1335_ = 30.64, *p*<.001; MBI = Moral Behavior Inventory; DSES = Daily Spiritual Experience Scale; IRI = Interpersonal Reactivity Index.

**Figure 2:**
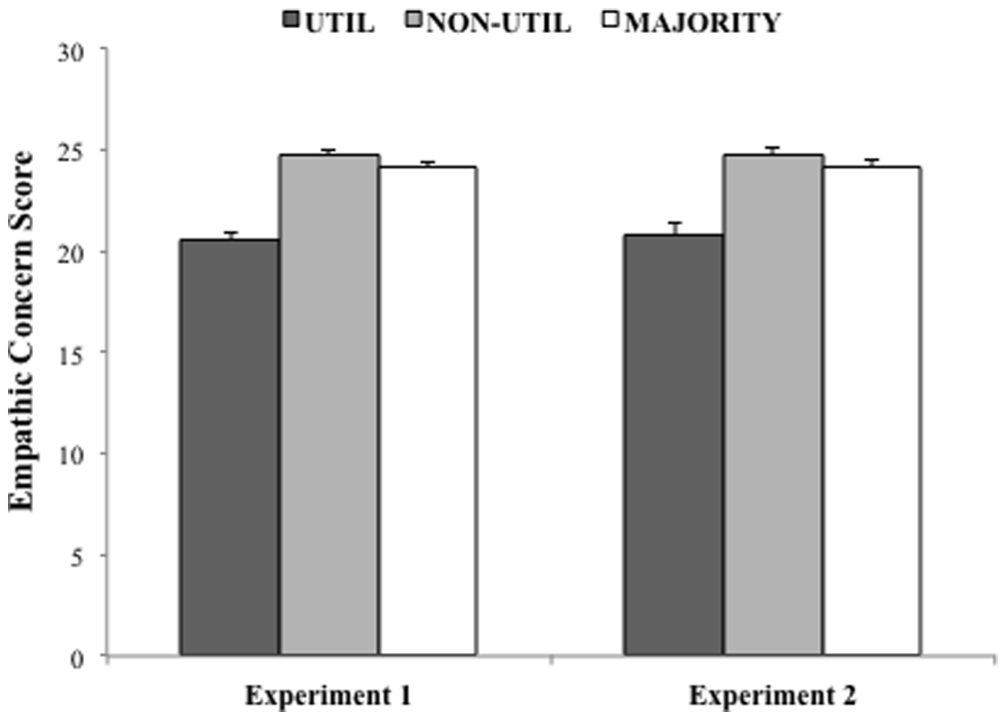
Empathic concern values for participants in the UTIL, NON-UTIL, and MAJORITY groups on the pairs of moral scenarios for Experiments 1 and 2. In both cases, UTIL participants had significantly lower empathic concern scores than participants in the NON-UTIL and MAJORITY groups. Error bars represent *S.E.M.*

However, differences between participant groups emerged for the IRI. While groups did not differ significantly on fantasy (*F*_3,1335_ = 2.23, *p* = .08), perspective taking (*F*_3,1335_ = 1.87, *p* = .13), or personal distress (*F*_3,1335_ = 0.29, *p* = .83), a significant difference was found for empathic concern (*F*_3,1335_ = 30.64, *p*<.001). Bonferroni post hoc comparisons revealed that UTIL participants showed significantly lower empathic concern (EC) than each of the other participant groups (Figure 2): NON-UTIL (*p*<.001), MAJORITY (*p*<.001), and OUTLIER (*p*<.01). No other pairwise differences were found (NON-UTIL vs. MAJORITY: *p* = .82; NON-UTIL vs. OUTLIER: *p* = .42; MAJORITY vs. OUTLIER: *p* = .33). We replicated these results in an analysis that excluded participants in the OUTLIER group (i.e., including only participants in the UTIL, NON-UTIL, and MAJORITY groups; *n* = 1324), see Text S1. Therefore, OUTLIER participants were excluded from further analyses.

#### Personal vs. impersonal scenarios

Next, we investigated the relationship between empathy and moral judgment in response to the impersonal and personal scenarios, separately (Table 2).

**Table 2:** *Mean* (*SD*) values for moral knowledge, religiosity, and empathy questionnaires for participants who responded “YES” or “NO”, independently, to each moral scenario from Experiment 1.

		Impersonal Scenario			Personal Scenario	
		Utilitarian Response*n* = 819	Non-Utilitarian Response*n* = 505		Utilitarian Response*n* = 213	Non-Utilitarian Response*n* = 1111
MBI		61.3 (10.7)	60.0 (12.2)	60.3 (11.8)		60.9 (11.2)
DSES		60.2 (20.5)	58.4 (22.6)	58.3 (22.1)		59.7 (21.2)
IRI	Perspective Taking	20.8 (5.2)	20.6 (5.2)	20.4 (5.6)		20.8 (5.2)
	Fantasy	19.3 (5.7)	18.9 (5.8)	18.5 (5.9)		19.3 (5.7)
	Empathic Concern	23.6 (6.0)	24.2 (5.5)	20.5 (6.4)		24.4 (5.5)
	Personal Distress	14.9 (4.6)	14.8 (4.7)	14.6 (4.8)		14.9 (4.6)

MBI = Moral Behavior Inventory; DSES = Daily Spiritual Experience Scale; IRI = Interpersonal Reactivity Index.

##### Impersonal scenario

819 (61.9%) participants delivered the utilitarian response (e.g., yes, flip the switch), and 505 (38.1%) participants delivered the non-utilitarian response (e.g., no, don’t flip the switch) to the standard trolley dilemma. The groups were comparable on their levels of religiosity/spirituality (*t*_1322_ = 1.49, *p* = .14, Cohen’s *d* = .08). Utilitarian responders scored higher on the Moral Behavior Inventory (MBI) than the non-utilitarian responders (*t*_1322_ = 1.94, *p* = .05, Cohen’s *d* = .11). This effect was small (*d* = .11) and did not replicate in Experiment 2, so it will not be discussed further. No significant differences between the utilitarian and non-utilitarian responders were found on any of the empathy subscales (perspective taking: *t*_1322_ = 0.70, *p* = .49, Cohen’s *d* = .04; fantasy: *t*_1322_ = 1.08, *p* = .28, Cohen’s *d* = .06; empathic concern: *t*_1322_ = -1.73, *p* = .08, Cohen’s *d* = .10; personal distress: *t*_1322_ = 0.06, *p* = .95, Cohen’s *d* <.01).

##### Personal scenario

213 (16.1%) participants delivered the utilitarian response (e.g., yes, push the man), and 1111 (83.9%) participants delivered the non-utilitarian response (e.g., no, don’t push the man). The groups were comparable on their religiosity (*t*_1322_ = −0.91, *p* = .36, Cohen’s *d* = .05) and moral knowledge (*t*_1322_ = −0.70, *p* = .49, Cohen’s *d* = .04). No significant differences between the utilitarian and non-utilitarian responders were found on the perspective taking (*t*_1322_ = −0.93, *p* = .35, Cohen’s *d* = .05), fantasy (*t*_1322_ = −1.75, *p* = .08, Cohen’s *d* = .10), or personal distress (*t*_1322_ = −0.77, *p* = .44, Cohen’s *d* = .04). However, consistent with the prior analyses over the scenario pair, utilitarian participants who endorsed pushing the man showed significantly lower levels of empathic concern than non-utilitarian responders (*t*_1322_ = −9.27, *p*<.001, Cohen’s *d = *0.51).

#### Discriminatory analysis

**Figure 3:**
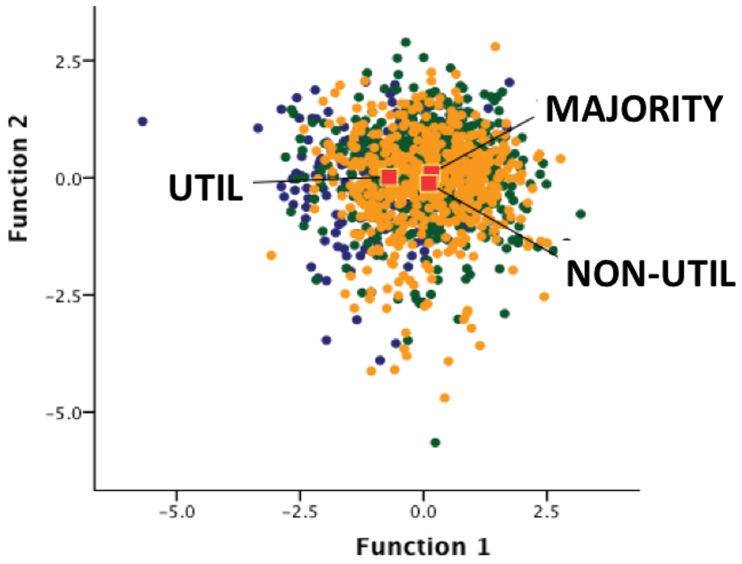
Distribution of UTIL (blue), DEON (orange), and MAJORITY (green) participants with regards to the two functions generated by discriminatory analysis. Red squares represent group centroids and reveal that UTIL participants are best distinguished from the other groups on the basis of function 1, for which empathic concern had the strongest load factor (see text).

A direct discriminant function analysis was performed using age, gender, level of education, religiosity, moral knowledge, and the 4 aspects of empathy as predictors of moral judgment profiles (i.e., UTIL, NON-UTIL or MAJORITY). Two discriminant functions were calculated, with a combined *χ* ^2^(18) = 132.0, p<.001, accounting for 90.7% and 9.3% of the between-group variance, respectively. As shown in Figure 3, Discriminant function 1 maximally separated UTIL (group centroid = −.70) from both NON-UTIL (group centroid = .11) and MAJORITY (group centroid = .16) participants and was statistically significant (*p*<.001, canonical correlation = .30). The second function maximally separated NON-UTIL (group centroid = −.12) from MAJORITY (group centroid = .10), but was not statistically significant (*p* = .12, canonical correlation = .09). The loading matrix of correlations of predictor variables and discriminant functions, as seen in Table 3, suggests that the primary variable in distinguishing UTIL from other participants was empathic concern (EC). See also Text S2 and Table S2.

**Table 3:** Loading matrix of correlations for discriminant analyses conducted in Experiments 1.

		Experiment 1	
Discriminatory Function		1	2
*p value* for function		<.001	n.s.
Age		−.17	.26
Gender		−.09	−.29
Education		.23	.03
MBI		.08	.63
DSES		.09	.51
IRI	Perspective Taking	.09	.28
	Fantasy	.17	.51
	Empathic Concern	**.84**	.22
	Personal Distress	.07	.08

Variables included in the leftmost column served as predictors in the classification of (1) UTIL from NON-UTIL and MAJORITY participants, and (2) NON-UTIL from MAJORITY participants. Empathic Concern was the factor that most strongly classified UTIL participants apart.

MBI = Moral Behavior Inventory; DSES = Daily Spiritual Experience Scale; IRI = Interpersonal Reactivity Index.

## Experiment 2

Experiment 2 served to replicate and extend the pattern observed in Experiment 1. We sought to determine whether the relationship between moral judgment and empathic concern would generalize beyond the trolley/footbridge pair of dilemmas, which differ along a number of dimensions [6]. Experiment 2 investigates the patterns observed above in an independent participant sample, using a new pair of moral scenarios, including a war-time “pareto” scenario (e.g., in which the individual person to be killed would die anyway). Experiment 2 therefore aims to investigate whether the results of Experiment 1 transfer to different moral contexts featuring different tradeoffs.

### Procedure

Experiment 2 followed the same procedures as Experiment 1. A new participant group [*n = *896; mean age: 24.8 years (*SD* = 15.7), mean education: 13.2 years (*SD* = 4.2)] was tested on a different pair of scenarios (Table S1). First, the standard fumes dilemma (impersonal scenario) asked whether it was morally permissible to redirect toxic fumes into one patient’s room to save the lives of three other patients. The utilitarian response was to flip the switch to redirect the fumes from three people and onto one person instead, whereas the non-utilitarian response was to allow the fumes to kill the three people. Second, the crying baby dilemma (personal scenario) asked whether it was morally permissible to smother a baby to death, while hiding during wartime, so that the soldiers would not hear the baby cry and kill everyone in hiding (including the baby). The utilitarian response was to smother the baby to save the others, whereas the non-utilitarian response was to let the baby cry, alerting the soldiers, resulting in many deaths. Relevant to the current hypothesis, the crying baby scenario (like the footbridge scenario in Experiment 1) pits one life against many lives in an emotionally salient (personal) context. We note that the crying baby scenario differs from the footbridge scenario of Experiment 1 in at least two respects. First, the crying baby scenario is a war-time (as opposed to peace-time) scenario. Second, the crying baby scenario is a “pareto” dilemma, since the one person who would be killed to save the other people would die no matter what the decision turned out to be [36]. The inclusion of the crying baby scenario allows us to explore preliminarily whether the pattern observed in Experiment 1 extends to war-time contexts and pareto tradeoffs.

As in Experiment 1, participants were classified into four groups based on their responses to the pair of scenarios: 117 (13.1%) UTIL, 249 (27.5%) NON-UTIL, 522 (58.3%) MAJORITY, and 11 (1.2%) OUTLIER.

### Results

#### UTIL vs. NON-UTIL comparison

Of chief importance, we replicated the key result of Experiment 1 (Figure 2): UTIL responders exhibited significantly lower empathic concern scores than NON-UTIL responders (*t*_361_ = −4.84, *p*<.001, Cohen’s *d = *0.51). Also as in Experiment 1, no significant differences were found for fantasy (*t*_361_ = −0.79, *p* = .43, Cohen’s *d* = .08), perspective taking (*t*_361_ = −0.81, *p* = .04, Cohen’s *d* = .09), or personal distress (*t*_361_ = −0.86, *p* = .58, Cohen’s *d* = .09). There was no difference between the UTIL and NON-UTIL groups in age (*t*_361_ = 1.25, *p* = .21, Cohen’s *d* = .13), gender (*χ* ^2^ = 0.21, *p* = .65), education (*t*_361_ = 0.31, *p* = .76, Cohen’s *d* = .03), moral knowledge (*t*_361_ = −0.09, *p* = .93, Cohen’s *d* <.01), or religiosity (*t*_361_ = −0.45, *p* = .66, Cohen’s *d* = .05) (Table 4). Similar results were found when including the MAJORITY group (all *p*>.13), and both the MAJORITY and OUTLIER groups (all *p*>.11) in the ANOVAs.

**Table 4:** *Mean* (*SD*) values for demographic variables and data obtained with the moral knowledge, religiosity, and empathy questionnaires for Experiment 2.

		Experiment 2	
		UTIL	NON-UTIL
Age (years)		26.5 (12.1)	24.9 (11.4)
Gender (M : F)		51∶66	101∶145
Education (years)		13.4 (3.8)	13.5 (4.1)
MBI		60.7 (12.2)	60.8 (11.7)
DSES		57.4 (21.8)	58.5 (22.1)
IRI	Perspective Taking	20.2 (5.8)	20.7 (4.9)
	Fantasy	18.1 (6.1)	18.6 (5.6)
	Empathic Concern	21.1 (6.2)	24.1 (5.6)^a^
	Personal Distress	14.5 (5.0)	15.0 (4.8)

a *t*_361_ = −4.84, *p*<.001; MBI = Moral Behavior Inventory; DSES = Daily Spiritual Experience Scale; IRI = Interpersonal Reactivity Index.

#### Personal vs. Impersonal scenarios

##### Impersonal scenario

546 (61.7%) participants delivered the utilitarian response (e.g., yes, redirect the fumes), and 339 (38.3%) participants delivered the non-utilitarian response (e.g., no, do not redirect the fumes). The groups were comparable on their levels of religiosity/spirituality (*t*_883_ = 0. 94, *p* = .35, Cohen’s *d* = .06) and moral knowledge (*t*_883_ = 0. 81, *p* = .42, Cohen’s *d* = .05). Replicating Experiment 1, no significant differences between the utilitarian and non-utilitarian responders were found on any of the empathy subscales (perspective taking: *t*_883_ = 0.51, *p* = .61, Cohen’s *d* = .03; fantasy: *t*_883_ = 0.94, *p* = .35, Cohen’s *d* = .06; empathic concern: *t*_883_ = −1.26, *p* = .21, Cohen’s *d* = .08; personal distress: *t*_883_ = −0.44, *p* = .66, Cohen’s *d* = .03).

##### Personal scenario

138 (15.6%) participants delivered the utilitarian response (e.g., yes, smother the baby), and 747 (84.4%) participants delivered the non-utilitarian response (e.g., no, don’t smother the baby). The groups were comparable on religiosity (*t*_883_ = 0.67, *p* = .50) and moral knowledge (*t*_883_ = −0.83, *p* = .41). No significant differences between the utilitarian and non-utilitarian responders were found on perspective taking (*t*_883_ = −1.33, *p* = .19, Cohen’s *d* = .07), fantasy (*t*_883_ = −1.82, *p* = .07, Cohen’s *d* = .10), or personal distress (*t*_883_ = −0.74, *p* = .46, Cohen’s *d* = .04). Crucially, though, replicating the key pattern in Experiment 1, participants who endorsed smothering the baby showed significantly lower levels of empathic concern (20.8±6.2) than non-utilitarian responders (24.5±5.7; *t*_883_ = −6.79, *p*<.001, Cohen’s *d = *0.46). See also Text S3 and Table S2.

## Experiment 3

Like Experiments 1 and 2, Experiment 3 also presented a pair of moral scenarios. This scenario pair included not only a moral dilemma, as in the prior experiments, but also a *prudential* dilemma featuring the choice to commit a moral transgression for one’s own selfish benefit (rather than for the greater good). The primary aim of Experiment 3 was to replicate the effects observed in Experiments 1 and 2, but a secondary aim was to explore whether low empathic concern is uniquely associated with utilitarian responses (i.e., harming one to save many), or whether low empathic concern is also associated with endorsing harmful acts across the board, including impersonal selfish acts (e.g., cheating on one’s taxes)?

### Procedure

An independent group of participants [*n = *513; mean age: 25.7 (*SD* = 12.9); mean education: 13.5 (*SD = *4.3)] responded to two new scenarios. The impersonal taxes scenario asked whether it was permissible to report personal expenses as business expenses in order to save money. Crucially, the taxes scenario [12], [13] is not a *moral dilemma* of the kind tested in Experiments 1 and 2, insofar as there is no clear utilitarian or non-utilitarian response. Instead, one response was to report personal expenses as business expenses - the execution of a plainly immoral, antisocial act for selfish benefit, whereas the other response was to report only the real business expenses - the morally right thing to do. In this sense, the taxes dilemma might be considered a *prudential dilemma* – should one act immorally for one’s own sake? A unique benefit of including the taxes scenario in Experiment 3 was to obtain a preliminary sense of whether utilitarian responders *and* plainly immoral agents alike are lower in empathic concern. Do utilitarians endorse harming one to save many simply because they endorse harmful, selfish acts more generally? Or, alternatively, as we propose, does reduced empathic concern lead *specifically* to utilitarian moral judgments?

Therefore, we also presented the same participants with a moral dilemma, much like the moral dilemmas in Experiments 1 and 2. The personal transplant dilemma asked whether it was permissible to transplant the organs of one patient, against his will, to save the lives of five patients. The utilitarian response was to perform the transplant, killing one but saving five, whereas the non-utilitarian response was to respect the patient’s wishes not to transplant his organs, letting the five die.

### Results

#### Prudential dilemma

**Figure 4:**
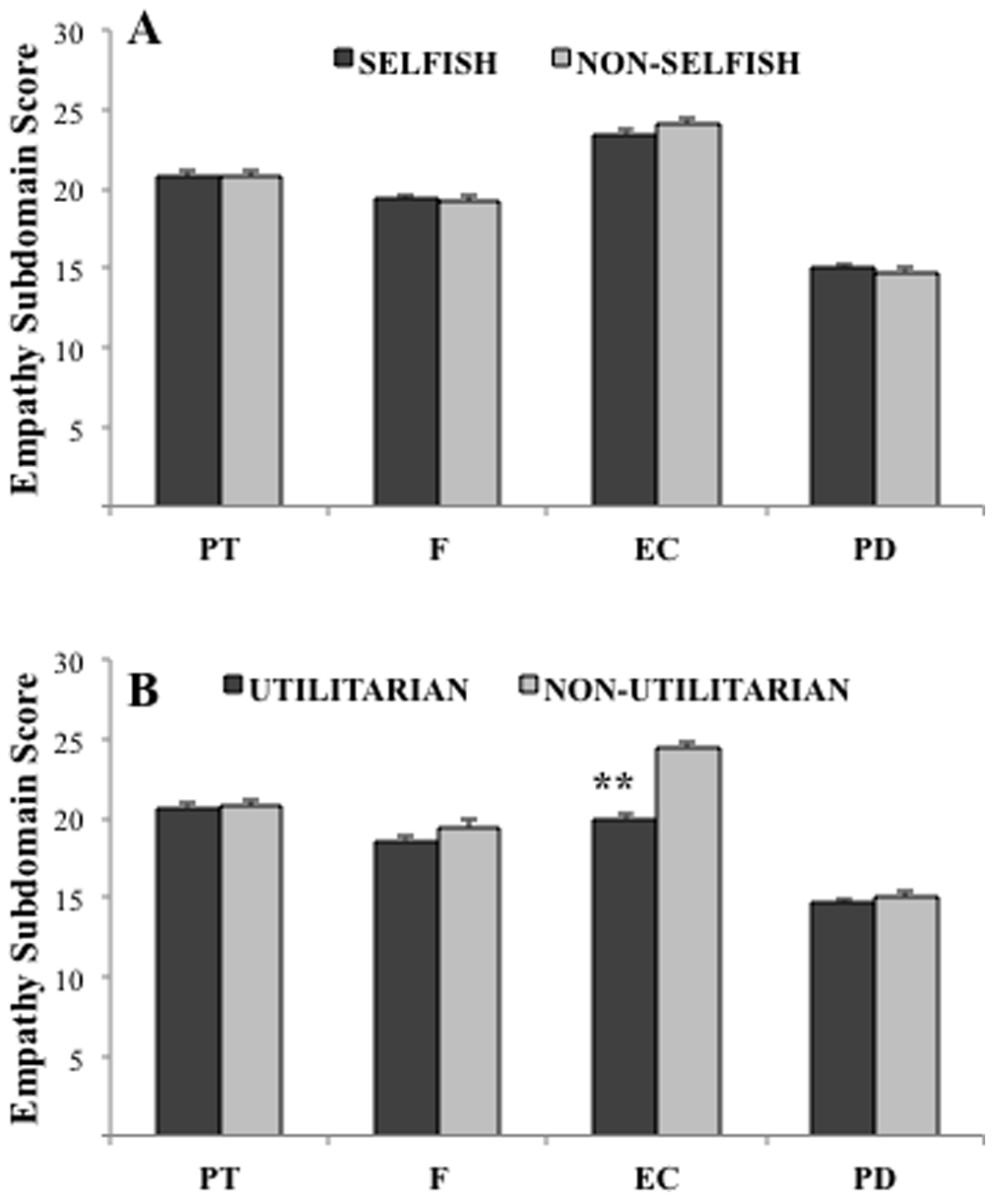
Scores obtained on the Perspective Taking (PT), Fantasy (F), Empathic Concern (EC), and Personal Distress (PD) subdomains of empathy for (A) selfish vs. non-selfish responses on the prudential taxes dilemma and (B) utilitarian vs. non-utilitarian responses on the personal transplant dilemma of Experiment 3. A significant difference (**p<.001) was exclusively found on empathic concern between utlitarian and non-utilitarian responders on the personal dilemma. Error bars represent *S.E.M.*

316 (62.2%) participants delivered the selfish response (e.g., yes, report personal expenses as work-related), and 192 (37.8%) participants delivered the non-selfish response (e.g., no, don’t report personal expenses as work-related). The groups were comparable in their religiosity/spirituality (*t*_506_ = 0. 64, *p* = .52, Cohen’s *d* = .06), although selfish responders (MBI *Score:* 61.3±58.7) scored higher than non-selfish responders (MBI *Score*: 58.7±13.2) on moral knowledge (*t*_506_ = 2.49, *p* = .01, Cohen’s *d* = .22). As shown by Figure 4, no significant differences between the groups were found on any of the empathy subscales (perspective taking: *t*_506_ = 0.14, *p* = .89, Cohen’s *d* = .01; fantasy: *t*_506_ = 0.35, *p* = .73, Cohen’s *d* = .03; empathic concern: *t*_506_ = −1.32, *p* = .19, Cohen’s *d* = .11; personal distress: *t*_506_ = 0.74, *p* = .46, Cohen’s *d* = .07).

#### Moral dilemma

87 (17.1%) participants delivered the utilitarian response (e.g., yes, proceed with the transplant), and 421 (82.9%) participants delivered the non-utilitarian response (e.g., no, don’t do the transplant). The groups were comparable on their religiosity (*t*_506_ = 0.82, *p* = .41, Cohen’s *d* = .07) and moral knowledge (*t*_506_ = 0.33, *p* = .74, Cohen’s *d* = .03). No significant differences between the utilitarian and non-utilitarian responders were found on the perspective taking (*t*_506_ = −0.15, *p* = .88, Cohen’s *d* = .01), fantasy (*t*_506_ = −1.22, *p* = .11), or personal distress (*t*_506_ = −0.58, *p* = .56, Cohen’s *d* = .05) domains of empathy. Finally, we replicated the key result of Experiments 1 and 2: participants who stated they would proceed with the transplant (i.e., the utilitarian response) showed significantly lower levels of empathic concern (19.9±6.6) than non-utilitarian responders (24.5±5.0; *t*_506_ = −7.18, *p*<.001, Cohen’s *d = *0.64).

## Discussion

An extensive body of prior research indicates an association between emotion and moral judgment. In the present study, we characterized the predictive power of specific aspects of emotional processing (e.g., empathic concern versus personal distress) for different kinds of moral responders (e.g., utilitarian versus non-utilitarian). Across three large independent participant samples, using three distinct pairs of moral scenarios, we observed a highly specific and consistent pattern of effects. First, moral judgment was uniquely associated with a measure of empathy but unrelated to any of the demographic or cultural variables tested, including age, gender, education, as well as differences in “moral knowledge” and religiosity. Second, within the complex domain of empathy, utilitarian judgment was consistently predicted only by *empathic concern*, an emotional component of empathic responding. In particular, participants who consistently delivered utilitarian responses for both personal and impersonal dilemmas showed significantly *reduced* empathic concern, relative to participants who delivered non-utilitarian responses for one or both dilemmas. By contrast, participants who consistently delivered *non-utilitarian* responses on both dilemmas did not score especially high on empathic concern or any other aspect of empathic responding.

### The Role of Demographic and Cultural Variables in Moral Judgment

The current study suggests no association between demographic or cultural variables and moral judgment of the kind probed in our study across three relatively large samples. Although some studies have documented the role of gender in certain aspects of moral judgment [37], [38], this effect appears to be mediated by differences in the emotional and empathic responding associated with sex and gender differences [39]–[41]. For example, females have been shown to more strongly endorse utilitarian judgments following administration of testosterone [42]. In addition, the present study included a measure of “moral gnosia” to determine whether differences in the way participants explicitly reason about right and wrong, in general terms, might influence participants’ judgments on specific moral dilemmas. Again, we found no relationship between “moral knowledge” as measured by the Moral Behavior Inventory and moral judgment, broadly consistent with prior research showing no relationship between moral judgment and education or religious belief [31], [37], [43]. The absence of any impact of demographic or cultural variables on moral judgment underscores the specific role of emotional responding in moral utilitarianism, as we discuss in detail below.

### The Role of Emotional Responding in Moral Judgment

On a dual-process theory of moral cognition, automatic emotional intuitions that support non-utilitarian judgments compete with controlled processes that support utilitarian judgments [10], [12], [13], [44]. Faced with a moral dilemma, people might experience a conflict between these two systems. Thus, utilitarian judgment could result from either enhanced cognitive control or abstract reasoning (i.e., to override prepotent emotional responses) or diminished emotional responses. Consistent with the former account, participants with greater working memory capacity were more likely to deliver utilitarian judgments on personal moral scenarios [45]. Moreover, under cognitive load, utilitarian decision-making was rendered slower [11], while non-utilitarian decision-making was unaffected. In fact, moral judgments are altered when cognitive control is impaired by manipulating response time, including when participants are forced to respond to a moral dilemma within seconds versus within minutes [28]. Finally, utilitarian versus non-utilitarian judgment elicited higher activity in anterior cingulate cortex and dorsolateral prefrontal cortex, brain regions associated with abstract reasoning and cognitive control [12]. These results suggest that utilitarians may be able to deliver utilitarian moral judgments primarily because of their greater cognitive control over gut emotional responses.

In conjunction with recent research, our findings support an alternative route to utilitarian moral decision-making in a neurotypical population and add important cognitive detail. Diminished emotional responses, specifically, reduced *empathic concern,* appear to be critical in facilitating utilitarian responses to moral dilemmas of high emotional salience. Recent findings, using behavioral priming methods, are consistent with this proposal. In one study, participants who viewed a humorous video before responding to the personal footbridge dilemma were more likely to endorse pushing the man off the footbridge [15]. Diminishing the negative emotional response and perhaps also the empathic concern for the potential victim via extraneous positive affect may have enabled utilitarian responding. Other work has identified that irreverence, specifically, rather than awe or elevation, leads to more utilitarian moral judgments [46]. In fact, utilitarian judgments are even predicted by participants’ level of emotional arousal: in recent work, utilitarian moral judgments were associated with lower autonomic arousal, as measured via electrodermal activity of skin conductance in response to moral scenarios [47], [48].

Convergent neuropsychological evidence also reveals that patient populations characterized by deficits in social emotions show abnormally utilitarian judgment. Patients with damage to the ventromedial prefrontal cortex (vmPFC) are more likely to endorse harming one to save many [18], [19]. Patients with behavioral variant frontotemporal dementia (bvFTD) are also more likely to deliver utilitarian judgments relative to patients with other dementias and healthy controls [20]. Notably, utilitarian responders within the bvFTD population show diminished performance specifically on tasks probing emotional empathy [49]. Extensive work supports the role of emotion in moral development as well as moral cognition at the mature state [50]–[52].

Individual differences in empathic concern may also interact with different cognitive and neural mechanisms for moral judgment. For instance, pharmacologically enhanced levels of serotonin, a neurotransmitter implicated in prosocial behavior, influence the moral judgment of individuals high in trait empathy, leading to more deontological moral judgment, whereas individuals low in trait empathy are relatively unaffected by altered serotonin levels [53]. Furthermore, a recent study by Conway and Gawronsky [54] has shed light on the differential influence of cognitive load and empathy enhancement on moral judgment. Asking participants to complete a working memory task while responding to moral dilemmas decreased utilitarian judgment but did not increase deontological responses. Meanwhile, enhancing empathic concern by showing participants a negatively valenced image in association with moral dilemmas (e.g., a picture of a crying baby) specifically increased deontological judgment, but did not decrease utilitarian responses. Thus, the dissociable effects of cognitive load and empathic concern on moral judgment provide additional evidence that two alternative routes may lead to utilitarian moral judgment: enhanced cognitive control, on the one hand, or, as proposed by the present findings, decreased empathic concern, on the other hand.

Building on this prior work, the present findings support an important alternative route to utilitarian judgment. Utilitarian judgment may arise not simply from enhanced cognitive control but also from diminished emotional processing and, in particular, reduced empathic concern. The convergence of the current results with previous research constitutes an important part of the present study, especially in light of recent reports showing the importance of reproducing results in validating findings [55]. Nevertheless, we also take the current results to reflect novel theoretical contributions that we describe in additional detail in the remaining two sections.

### Are Utilitarians Simply Antisocial?

The present findings are consistent with recent behavioral work revealing that utilitarian responders exhibit traits typically associated with diminished emotional reactivity. Recent work by Bartels and Pizarro [3] found that participants endorsing utilitarian judgments to personal moral dilemmas scored higher on measures of antisocial personality. The current study lends further support and important cognitive detail to this behavioral pattern. First, the current findings rely on a different measure of emotional responding, specifically, for assessing empathy. Second, we found no relationship between moral judgment and participants’ scores on other key domains of empathy, i.e. personal distress, perspective taking, or fantasy, highlighting the specificity of the relationship between moral judgment and *empathic concern* in the present paradigm. Third, the current results demonstrate that the “opposite” pattern (e.g., enhanced empathic responding) does not describe consistently non-utilitarian participants. That is, individuals who showed especially *non-utilitarian* patterns of judgment did not score higher on any measure of empathy. Future work should target more directly the psychological determinants of non-utilitarian responding.

It is also worth noting that differences in empathy (including empathic concern) did not predict the likelihood of endorsing a plainly immoral act in Experiment 3, i.e., cheating on one’s taxes, typically associated with antisocial personality or psychopathy [56]. In other words, the actions of immoral agents and moral utilitarians were not equivalently determined by empathic concern in the current experimental context. Of course, additional work comparing personal immoral acts to personal utilitarian acts is required; however, these preliminary data suggest that reduced empathic concern may lead to utilitarian moral judgments specifically, and not to simply immoral or selfish antisocial acts in general. Indeed, because of its *other-oriented* nature, empathic concern seems to be elicited principally when harm is inflicted on a third-party victim, consistent with prior work demonstrating the asymmetric impact of empathy on altruism and pro-social behavior but not simply selfish or self-focused behavior [57], [58]. It is important to note that the prudential dilemma presented in Experiment 3 is *impersonal*. The high proportion of selfish responders in our current sample compared with previous reports is worthy of further research; this pattern might reflect culture-specific attitudes toward selfish transgressions that do not lead to harm towards specific targets (i.e., cheating in one’s taxes for personal benefit) or, in addition, differences in explicit cultural norms regarding taxes. In an exploratory analysis, we have investigated the relationship between selfish tendencies and endorsement of the utilitarian option in the personal moral scenario; in brief, participants who reported that they would not cheat on their taxes also reported that they did not endorse the utilitarian option (Table S3). Future work should also investigate whether *personal* prudential dilemmas elicit the same pattern as personal moral dilemmas. Future work should examine whether reduced empathic concern also leads to plainly immoral personal behavior.

### The Specific Role of Empathic Concern

The absence of any association between utilitarian moral judgment and any other aspect of empathy in the current study might be surprising given prior accounts. On one account, the affective state elicited in the observer in response to another person’s emotions or experience might lead not only to feelings of warmth and compassion *for* the target of empathy (empathic concern) but also to self-centered feelings of discomfort triggered *by* the target (personal distress) [59]. However, personal distress did not predict utilitarian moral judgment in any of the three experiments. This result suggests that “extreme utilitarians” differ from the average respondent not in their affective state as a whole, but, rather, in the specific set of emotions that may be elicited *for* an agent (e.g., empathic concern). Relatedly, the present behavioral pattern suggests it is unlikely for the apparent “hypoaffective state” to result from an enhanced ability to regulate emotions *in general*; otherwise, we might have seen the same effect for personal distress. That is, utilitarian responders might have exhibited not only reduced empathic concern but also reduced personal distress. It is important to note that this specific effect of empathic concern rules out the possibility that participants who had originally reported higher values on the empathy scale were trying to be consistent in their responses to moral scenarios; consistency bias should not apply specifically to empathic concern and not, for example, personal distress or perspective taking. Moreover, IRI items were not presented in clusters by component. And, finally, the order of questionnaires (e.g., moral scenarios, IRI, etc.) was randomized across participants.

Importantly, moral judgments were not predicted by differences in perspective taking ability either. On one account, if an observer were better able to take the perspective of another person, i.e. the victim, the observer might experience a stronger emotional response to the victim’s pain or distress [60]. However, the current study demonstrates that utilitarian responders may be as capable at perspective taking as non-utilitarian responders. As such, utilitarian moral judgment appears to be specifically associated with a diminished affective reactivity to the emotions of others (empathic concern) that is independent of one’s ability for perspective taking, supporting also the differential effects of empathic concern and perspective taking in social cognition [29], [61], [62].

### Conclusions

Utilitarian moral judgment in the current study was specifically associated with reduced empathy and not with any of the demographic or cultural variables tested. Moreover, utilitarian moral judgment was determined uniquely by levels of empathic concern, independent of other aspects of empathic responding including personal distress and perspective taking. Levels of empathic concern in “extreme utilitarians” (but not “extreme non-utilitarians”) deviated from the majority of responders. Diminished levels of emotional responding may therefore enable moral utilitarians to consistently favor harmful actions that maximize aggregate welfare. Indeed, how we resolve moral dilemmas may rely not simply on abstract reasoning and cognitive control but also crucially on our empathic concern for potential victims.

## Supporting Information

Table S1 (DOC)
Pairs of impersonal/personal moral dilemmas. Adapted from Greene et al. (2004).
(DOC)

Table S2 Number of participants who showed low (low-EC) or high (high-EC) empathic concern grouped according to their responses on the impersonal and personal scenarios, and dilemma pair.
(DOC)

Table S3 Analysis of selfish responses and moral personal responses in Experiment 3. Participants who reported that they would not cheat on their taxes also reported that they did not endorse the utilitarian option.
(DOC)

Text S1 **Analyses performed excluding the OUTLIER group.**
(DOC)

Text S2 **Comparison of High vs. Low Empathic Concern in Experiment 1.**
(DOC)

Text S3 **Comparison of High vs. Low Empathic Concern in Experiment 2.**
(DOC)

## References

[1] Mikhail J (2007) Universal moral grammar: theory, evidence and the future. Trends Cogn Sci 11: 143–152. 17329147 10.1016/j.tics.2006.12.007

[2] Schaich Borg J, Hynes C, Van Horn J, Grafton S, Sinnott-Armstrong W (2006) Consequences, action, and intention as factors in moral judgments: an FMRI investigation. J Cogn Neurosci 18: 803–817. 16768379 10.1162/jocn.2006.18.5.803

[3] Bartels DM, Pizarro DA (2011) The mismeasure of morals: Antisocial personality traits predict utilitarian responses to moral dilemmas. Cognition 121: 154–161. 21757191 10.1016/j.cognition.2011.05.010

[4] Nichols S (2002) Norms with feeling: towards a psychological account of moral judgment. Cognition 84: 221–236. 12175573 10.1016/s0010-0277(02)00048-3

[5] Inbar Y, Pizarro DA, Bloom P (2012) Disgusting smells cause decreased liking of gay men. Emotion (Washington, DC) 12: 23–27. 10.1037/a0023984 21707161

[6] Nichols S, Mallon R (2006) Moral dilemmas and moral rules. Cognition 100: 530–542. 16157325 10.1016/j.cognition.2005.07.005

[7] Blair RJ (1995) A cognitive developmental approach to mortality: investigating the psychopath. Cognition 57: 1–29. 7587017 10.1016/0010-0277(95)00676-p

[8] Prinz J (2006) The emotional basis of moral judgments. Philosophical Explorations 9: 29–43.

[9] Prinz J (2008) The Emotional Construction of Morals. New York: Oxford University Press.

[10] Greene J (2003) From neural “is” to moral “ought”: what are the moral implications of neuroscientific moral psychology? Nat Rev Neurosci 4: 846–849. 14523384 10.1038/nrn1224

[11] Greene J, Morelli SA, Lowenberg K, Nystrom LE, Cohen JD (2008) Cognitive load selectively interferes with utilitarian moral judgment. Cognition 107: 1144–1154. 18158145 10.1016/j.cognition.2007.11.004 PMC2429958

[12] Greene J, Nystrom LE, Engell AD, Darley JM, Cohen JD (2004) The neural bases of cognitive conflict and control in moral judgment. Neuron 44: 389–400. 15473975 10.1016/j.neuron.2004.09.027

[13] Greene J, Sommerville RB, Nystrom LE, Darley JM, Cohen JD (2001) An fMRI investigation of emotional engagement in moral judgment. Science 293: 2105. 11557895 10.1126/science.1062872

[14] Haidt J (2001) The emotional dog and its rational tail: a social intuitionist approach to moral judgment. Psychol Rev 108: 814–834. 11699120 10.1037/0033-295x.108.4.814

[15] Valdesolo P, DeSteno D (2006) Manipulations of emotional context shape moral judgment. Psychol Sci 17: 476–477. 16771796 10.1111/j.1467-9280.2006.01731.x

[16] Greene J, Cushman FA, Stewart LE, Lowenberg K, Nystrom LE, et al (2009) Pushing moral buttons: the interaction between personal force and intention in moral judgment. Cognition 111: 364–371. 19375075 10.1016/j.cognition.2009.02.001

[17] Cushman F, Gray K, Gaffey A, Mendes W (2012) Simulating murder: the aversion to harmful action. Emotion 12: 2–7. 21910540 10.1037/a0025071

[18] Ciaramelli E, Muccioli M, Ladavas E, Di Pellegrino G (2007) Selective deficit in personal moral judgment following damage to ventromedial prefrontal cortex. Soc Cogn Affect Neurosci 2: 84–92. 18985127 10.1093/scan/nsm001 PMC2555449

[19] Koenigs M, Young L, Adolphs R, Tranel D, Cushman F, et al (2007) Damage to the prefrontal cortex increases utilitarian moral judgements. Nature 446: 908–911. 17377536 10.1038/nature05631 PMC2244801

[20] Mendez MF, Anderson E, Shapira JS (2005) An investigation of moral judgement in frontotemporal dementia. Cognitive and Behavioral Neurology: Official Journal of the Society for Behavioral and Cognitive Neurology 18: 193–197. 16340391 10.1097/01.wnn.0000191292.17964.bb

[21] Koven NS (2011) Specificity of meta-emotion effects on moral decision-making. Emotion (Washington, DC) 11: 1255–1261. 10.1037/a0025616 21942703

[22] Wheatley T, Haidt J (2005) Hypnotic disgust makes moral judgments more severe. Psychol Sci 16: 780–784. 16181440 10.1111/j.1467-9280.2005.01614.x

[23] Eskine KJ, Kacinik NA, Prinz JJ (2011) A Bad Taste in the Mouth: Gustatory Disgust Influences Moral Judgment. Psychol Sci 22: 295–299. 21307274 10.1177/0956797611398497

[24] Schnall S, Haidt J, Clore GL, Jordan AH (2008) Disgust as embodied moral judgment. Pers Soc Psychol Bull 34: 1096–1109. 18505801 10.1177/0146167208317771 PMC2562923

[25] Inbar Y, Pizarro D, Knobe J, Bloom P (2009) Disgust sensitivity predicts intuitive disapproval of gays. Emotion 9: 435–439. 19485621 10.1037/a0015960

[26] Inbar Y, Pizarro D, Bloom P (2008) Conservatives are more easily disgusted than liberals. Cognition and Emotion 23: 714.

[27] Inbar Y, Pizarro D, Iyer R, Haidt J (2012) Disgust sensitivity, political conservatism, and voting. Social Psychological and Personality Science 3: 537–544.

[28] Suter R, Hertwig R (2011) Time and moral judgment. Cognition 119: 454–458. 21354557 10.1016/j.cognition.2011.01.018

[29] Batson DC (2009) These things called empathy: eight related but distinct phenomena. In: Decety J, Ickes WJ, editors. The Social Neuroscience of Empathy. Cambridge, MA: The MIT Press.

[30] Cushman F, Young L, Hauser M (2006) The role of conscious reasoning and intuition in moral judgment: testing three principles of harm. Psychol Sci 17: 1082–1089. 17201791 10.1111/j.1467-9280.2006.01834.x

[31] Hauser M, Cushman F, Young L, Jin R, Mikhail J (2007) A Dissociation Between Moral Judgments and Justifications. Mind and Language 22: 1–21.

[32] Underwood LG, Teresi JA (2002) The daily spiritual experience scale: development, theoretical description, reliability, exploratory factor analysis, and preliminary construct validity using health-related data. Ann Behav Med 24: 22–33. 12008791 10.1207/S15324796ABM2401_04

[33] Davis MH (1983) Measuring individual differences in empathy: Evidence for a multidimensional approach. Journal of Personality and Social Psychology 44: 113–126.

[34] Iyer R, Koleva S, Graham J, Ditto P, Haidt J (2012) Understanding libertarian morality: The psychological dispositions of self-identified libertarians. PloS one 7: e42366. 22927928 10.1371/journal.pone.0042366 PMC3424229

[35] Cohen J (1992) Statistical power analysis. Current directions in psychological science 1: 98–101.

[36] Huebner B, Hauser M, Pettit P (2011) How the Source, Inevitability and Means of Bringing About Harm Interact in Folk-Moral Judgments. Mind & Language 26: 210–233.

[37] Fumagalli M, Ferrucci R, Mameli F, Marceglia S, Mrakic-Sposta S, et al (2010) Gender-related differences in moral judgments. Cogn Process 11: 219–226. 19727878 10.1007/s10339-009-0335-2

[38] Harenski CL, Antonenko O, Shane MS, Kiehl KA (2008) Gender differences in neural mechanisms underlying moral sensitivity. Soc Cogn Affect Neurosci 3: 313–321. 19015084 10.1093/scan/nsn026 PMC2607058

[39] Baron-Cohen S, Wheelwright S (2004) The empathy quotient: an investigation of adults with Asperger syndrome or high functioning autism, and normal sex differences. J Autism Dev Disord 34: 163–175. 15162935 10.1023/b:jadd.0000022607.19833.00

[40] Fumagalli M, Vergari M, Pasqualetti P, Marceglia S, Mameli F, et al (2010) Brain switches utilitarian behavior: does gender make the difference? PLoS One 5: e8865. 20111608 10.1371/journal.pone.0008865 PMC2810338

[41] Jaffee S, Hyde JS (2000) Gender differences in moral orientation: a meta-analysis. Psychol Bull 126: 703–726. 10989620 10.1037/0033-2909.126.5.703

[42] Montoya ER, Terburg D, Bos PA, Will G-J, Buskens V, et al.. (2013) Testosterone administration modulates moral judgments depending on second-to-fourth digit ratio. Psychoneuroendocrinology. doi:10.1016/j.psyneuen.2012.12.001. 10.1016/j.psyneuen.2012.12.001 23290991

[43] Petrinovich L (1995) Human Evolution, Reproduction and Morality. New York: Plenum Press.

[44] Greenwald AG, Nosek BA, Banaji MR (2003) Understanding and using the Implicit Association Test: I. An improved scoring algorithm. Journal of Personality and Social Psychology 85: 197–216. 12916565 10.1037/0022-3514.85.2.197

[45] Moore AB, Clark BA, Kane MJ (2008) Who shalt not kill? Individual differences in working memory capacity, executive control, and moral judgment. Psychol Sci 19: 549–557. 18578844 10.1111/j.1467-9280.2008.02122.x

[46] Strohminger N, Lewis RL, Meyer DE (2011) Divergent effects of different positive emotions on moral judgment. Cognition 119: 295–300. 21255773 10.1016/j.cognition.2010.12.012

[47] Moretto G, Làdavas E (2010) A psychophysiological investigation of moral judgment after ventromedial prefrontal damage. J Cogn Neurosci 22: 1888–1899. 19925181 10.1162/jocn.2009.21367

[48] Navarrete CD, McDonald MM, Mott ML, Asher B (2011) Virtual morality: Emotion and action in a simulated three-dimensional “trolley problem”. Emotion 12: 364–370. 22103331 10.1037/a0025561

[49] Gleichgerrcht E, Torralva T, Roca M, Pose M, Manes F (2011) The role of social cognition in moral judgment in frontotemporal dementia. Social neuroscience 6: 113–122. 20706963 10.1080/17470919.2010.506751

[50] Decety J, Howard LH (2013) The Role of Affect in the Neurodevelopment of Morality. Child Development Perspectives 7: 49–54.

[51] Decety J, Cacioppo S (2012) The speed of morality: a high-density electrical neuroimaging study. Journal of neurophysiology 108: 3068–3072. 22956794 10.1152/jn.00473.2012

[52] Decety J, Michalska KJ, Kinzler KD (2012) The contribution of emotion and cognition to moral sensitivity: a neurodevelopmental study. Cerebral cortex (New York, NY: 1991) 22: 209–220. 10.1093/cercor/bhr111 21616985

[53] Crockett MJ, Clark L, Hauser MD, Robbins TW (2010) Serotonin selectively influences moral judgment and behavior through effects on harm aversion. Proceedings of the National Academy of Sciences of the United States of America 107: 17433–17438. 20876101 10.1073/pnas.1009396107 PMC2951447

[54] Conway P, Gawronski B (2013) Deontological and utilitarian inclinations in moral decision making: A process dissociation approach. Journal of personality and social psychology 104: 216–235. 23276267 10.1037/a0031021

[55] Simmons J, Nelson L, Simonsohn U (2011) False-Positive Psychology Undisclosed Flexibility in Data Collection and Analysis Allows Presenting Anything as Significant. Psychological Science 22: 1359–1366. 22006061 10.1177/0956797611417632

[56] Hare RD (1993) Without Conscience. New York: Guilford Press.

[57] Feldman Hall O, Dalgleish T, Thompson R, Evans D, Schweizer S, et al (2012) Differential neural circuitry and self-interest in real vs hypothetical moral decisions. Social cognitive and affective neuroscience 7: 743–751. 22711879 10.1093/scan/nss069 PMC3475363

[58] Van Lange PAM (2008) Does empathy trigger only altruistic motivation? How about selflessness or justice? Emotion (Washington, DC) 8: 766–774. 10.1037/a0013967 19102587

[59] Hodges SD, Biswas-Diener R (2007) Balancing the empathy expense account: strategies for regulating empathic response. In: Farrow TFD, Woodruff PWR, editors. Empathy in Mental Illness. Cambridge: Cambridge Uniersity Press. 389–405.

[60] Lamm C, Batson CD, Decety J (2007) The neural substrate of human empathy: effects of perspective-taking and cognitive appraisal. J Cogn Neurosci 19: 42–58. 17214562 10.1162/jocn.2007.19.1.42

[61] Gilin D, Maddux W, Carpenter J, Galinsky A (2013) When to use your head and when to use your heart: The differential value of perspective-taking versus empathy in competitive interactions. Pers Soc Psychol Bull 39: 3–16. 23150199 10.1177/0146167212465320

[62] Galinsky A, Maddux W (2008) Why It Pays to Get Inside the Head of Your Opponent The Differential Effects of Perspective Taking and Empathy in Negotiations. Psychol Sci 19: 378–384. 18399891 10.1111/j.1467-9280.2008.02096.x

