# Supplementary material for: Low Levels of Empathic Concern Predict Utilitarian Moral Judgment
Source: PLoS One. 2013 Apr 4;8(4):e60418. doi: 10.1371/journal.pone.0060418 (PMC3617220; doi:10.1371/journal.pone.0060418)
Supplement: Table S1 — (DOC) Pairs of impersonal/personal moral dilemmas. Adapted from Greene et al. (2004). (DOC) [file pone.0060418.s001.doc]

| Experiment | Scenario  Type | Scenario  Name | Scenario Text | Majority Response |
| --- | --- | --- | --- | --- |
| **Experiment 1** | Moral Impersonal | Trolley | Imagine that you are at the wheel of a runaway trolley quickly approaching a fork in the tracks. On the tracks going to the left is a group of five railway workmen. On the tracks going to the right is a single railway workman. If you do nothing, the trolley will proceed to the left, causing the deaths of the five workmen. The only way to avoid the deaths of these workmen is to hit a switch on your dashboard that will cause the trolley to proceed to the right, causing the death of the single workman.  *Would you hit the switch in order to avoid the deaths of the five workmen?* | YES |
|  | Moral Personal | Footbridge | Imagine that a runaway trolley is heading down the tracks toward five workmen who will be killed if the trolley keeps going. You are on a footbridge over the tracks in between the approaching trolley and the five workmen. Next to you on this footbridge is a stranger who happens to be very large. The only way to save the lives of the five workmen is to push this stranger off the bridge and onto the tracks below where his large body will stop the trolley. The stranger will die if you do this, but the five workmen will be saved.  *Would you push the stranger onto the tracks in order to save the five workmen?* | NO |
| **Experiment 2** | Moral Impersonal | Fumes | You are the night watchman in a hospital. There is an accident and deadly fumes in the ventilation system are traveling to the hospital rooms. In one room there are three patients. In another room there is a single man. If you do nothing the fumes will go into the room with the three patients and kill them. The only way to avoid this is to hit a switch that will reroute the fumes into the other room with the single man, killing him instead.  *Would you hit the switch in order to avoid the deaths of the three patients?* | YES |
|  | Moral  Personal | Crying  baby | Enemy soldiers have taken over your village. They have orders to kill everyone. You have hidden in the basement of a large house along with other people. Outside you hear the voices of soldiers who have come to search the house. Your baby begins to cry loudly. You cover his mouth to block the sound. If you remove your hand from his mouth the soldiers will hear his crying and will kill you, your baby, and the other people hiding in the basement. To save yourself and the others you must smother your child to death.  *Would you smother your child in order to save yourself and the other people?* | NO |
| **Experiment 3** | Prudential Impersonal | Taxes | You are the owner of a small business worried about surviving, making ends meet, and paying your taxes. It occurs to you that you can lower your taxes by pretending that some of your personal expenses are business expenses. For example, you could pretend that the stereo in your home is being used in your business office or that your dinners out with your wife are dinners with clients.  *Would you report personal expenses as business expenses in order to survive and make ends meet?* | YES |
|  | Moral Personal | Transplant | You are a doctor. You have five patients, each of whom is about to die due to a failing organ of some kind. You have another patient, a young man who is healthy. The only way that you can save the lives of the first five patients is to transplant five of this young man’s organs (against his will) into the bodies of the other five patients. If you do this, the young man will die, but the other five patients will live. The families and staff beg you to do this.  *Would you refuse their requests to do the transplants that would save five of your patients?* | NO |
