# Supplementary material for: Low Levels of Empathic Concern Predict Utilitarian Moral Judgment
Source: PLoS One. 2013 Apr 4;8(4):e60418. doi: 10.1371/journal.pone.0060418 (PMC3617220; doi:10.1371/journal.pone.0060418)
Supplement: Table S2 — Number of participants who showed low (low-EC) or high (high-EC) empathic concern grouped according to their responses on the impersonal and personal scenarios, and dilemma pair. (DOC) [file pone.0060418.s002.doc]

|  | | Experiment 1 | | |  | Experiment 2 | | |
| --- | --- | --- | --- | --- | --- | --- | --- | --- |
|  | | low-EC | high-EC | *Total* |  | low-EC | high-EC | *Total* |
| Impersonal Scenario | Utilitarian  response | 391 | 428 | 819 |  | 267 | 279 | 546 |
|  | Non-Utilitarian response | 223 | 282 | 505 |  | 147 | 192 | 339 |
|  |  |  |  |  |  |  |  |  |
| Personal  Scenario | Utilitarian  response | 140 | 73 | 213 |  | 91 | 47 | 138 |
|  | Non-Utilitarian response | 474 | 637 | 1111 |  | 323 | 424 | 747 |
|  | |  |  |  |  |  |  |  |
| Impersonal /Personal Pair of Scenarios | UTIL | 140 | 73 | 213 |  | 91 | 47 | 138 |
|  | MAJORITY | 251 | 355 | 606 |  | 176 | 232 | 408 |
|  | NON-UTIL | 223 | 282 | 505 |  | 147 | 192 | 339 |
|  | *Total* | 614 | 710 |  |  | 414 | 471 | 885 |
