# Supplementary material for: Low Levels of Empathic Concern Predict Utilitarian Moral Judgment
Source: PLoS One. 2013 Apr 4;8(4):e60418. doi: 10.1371/journal.pone.0060418 (PMC3617220; doi:10.1371/journal.pone.0060418)
Supplement: Table S3 — Analysis of selfish responses and moral personal responses in Experiment 3. Participants who reported that they would not cheat on their taxes also reported that they did not endorse the utilitarian option. (DOC) [file pone.0060418.s003.doc]

|  | | Personal Moral Scenario | |
| --- | --- | --- | --- |
|  | | Utilitarian | Non-Utilitarian |
| Impersonal Prudential Scenario | Selfish | 87 | 229 |
|  | Non-selfish | 0 | 192 |
