# Supplementary material for: Low Levels of Empathic Concern Predict Utilitarian Moral Judgment
Source: PLoS One. 2013 Apr 4;8(4):e60418. doi: 10.1371/journal.pone.0060418 (PMC3617220; doi:10.1371/journal.pone.0060418)
Supplement: Text S1 — Analyses performed excluding the OUTLIER group. (DOC) [file pone.0060418.s004.doc]

In order to determine whether further analyses could avoid the inclusion of a group that represented 1% of the respondents, we conducted between-group comparisons considering UTIL, NON-UTIL, and MAJORITY participants. We found no differences (all *p* > .05) in demographic or cultural variables [age (*F*_3,1321_ = 2.2), gender (*χ*^2^ = 2.26), moral knowledge (*F*_3,1321_ = 1.72), or religiosity (*F*_3,1321_ = 2.3)] (Table 1 in main text). Again, while groups did not differ significantly on fantasy (*F*_3,1321_ = 3.1, *p* = .07), perspective taking (*F*_3,1321_ = 1.03, *p* = .34), or personal distress (*F*_3,1321_ = 0.36, *p* = .70), a significant difference was found for empathic concern (*F*_3,1321_ = 44.2, *p* < .001). Bonferroni post hoc comparisons revealed that UTIL participants showed significantly lower empathic concern (EC) than both NON-UTIL (*p* < .001) and MAJORITY (*p* < .001) groups, while the latter two exhibited no pairwise differences (*p* = .41).
