# Supplementary material for: Low Levels of Empathic Concern Predict Utilitarian Moral Judgment
Source: PLoS One. 2013 Apr 4;8(4):e60418. doi: 10.1371/journal.pone.0060418 (PMC3617220; doi:10.1371/journal.pone.0060418)
Supplement: Text S2 — Comparison of High vs. Low Empathic Concern in Experiment 1. (DOC) [file pone.0060418.s005.doc]

We classified participants into “high” (high-EC) or “low” (low-EC) empathic concern groups, depending on whether their individual score for this subscale was above or below the sample’s mean (23.7, *SD* = 5.9), respectively. Of the 1339 participants included for this analysis, 625 (46.7%) scored below (low-EC), and 714 (53.3%) scored above (high-EC) the mean (Supplementary Table 2). Low-EC and high-EC participants did not differ in how they judged the impersonal moral scenario (*χ*^2^ = 1.63, *p* = .20, *df* = 1). However, a significantly higher proportion of participants in the high-EC (22.8%) versus the low-EC (10.3%) group responded “NO” on the personal scenario (*χ*^2^ = 38.1, *p* < .001, *df* = 1), the non-utilitarian response. The proportion of low-EC (65.7%) participants in the UTIL group was significantly higher (*χ*^2^ = 39.1, *p* < .001, *df* = 3) than in either the NON-UTIL (58.6%) or the MAJORITY (55.8%) groups (Table S2).
