# Supplementary material for: Low Levels of Empathic Concern Predict Utilitarian Moral Judgment
Source: PLoS One. 2013 Apr 4;8(4):e60418. doi: 10.1371/journal.pone.0060418 (PMC3617220; doi:10.1371/journal.pone.0060418)
Supplement: Text S3 — Comparison of High vs. Low Empathic Concern in Experiment 2. (DOC) [file pone.0060418.s006.doc]

Taking UTIL, DEON, and MAJORITY participants together, the *Mean* (*SD*) empathic concern for Experiment 2 (*n* = 885) was 23.9 (5.9). Of these, 414 (46.8%) respondents showed below-average empathic concern levels (low-EC) and 471 (53.2%) exhibited above-average empathic concern levels (high-EC). We replicated the results of Experiment 1 (Supplementary Table 3): for the impersonal scenario, the proportion of “YES” (utilitarian) and “NO” (deontological) responses was not significantly different for the high-EC and low-EC respondents (*χ*^2^ = 2.57, *p* = .11); however, for the personal moral scenario, more “YES” (utilitarian) responses emerged in the low-EC group (*χ*^2^ = 24.1, *p* < .001) (Table S2).
